# Optimizing Recovery Strategies in Elite Speedskating: A Comparative Analysis of Different Modalities

**DOI:** 10.3390/jfmk10010034

**Published:** 2025-01-16

**Authors:** Tomasz Kowalski, Kinga Rębiś, Jadwiga Malczewska-Lenczowska, Andrzej Klusiewicz, Michał Starczewski, Sebastian Klich, Przemysław Kasiak

**Affiliations:** 1 Department of Physiology, Institute of Sport—National Research Institute, 01-982 Warsaw, Poland; 2 Department of Nutrition Physiology, Institute of Sport—National Research Institute, 01-982 Warsaw, Poland; 3 Department of Physical Education and Health in Biala Podlaska, Faculty in Biała Podlaska, Jozef Pilsudski University of Physical Education, 00-968 Warsaw, Poland; 4 Faculty of Rehabilitation, Jozef Pilsudski University of Physical Education, 00-968 Warsaw, Poland; 5 Department of Sport Didactics, Wrocław University of Health and Sport Sciences, 51-612 Wroclaw, Poland; 6 3rd Department of Internal Medicine and Cardiology, Medical University of Warsaw, 02-091 Warsaw, Poland

**Keywords:** active recovery, compression boots, recovery breathing, short track

## Abstract

**Background/Objectives:** As short-track speed skaters have to race multiple races to achieve success during competition, optimizing the recovery between efforts is a noteworthy performance determinant. Therefore, we compared three different recovery modalities (active cycling recovery, pneumatic compression boots, and isocapnic breathing protocol) in the context of perceived subjective pain and recovery variables, multiple biochemical and biomechanical indices, CMJ height and power, as well as repeated efforts on the ice track. **Methods:** Fifteen elite short-track speed skaters (eight males and seven females; age 18.3 ± 1.0 years, height 175.6 ± 7.5 cm, weight 73.7 ± 7.7 kg, 23.8 kg/m^2^, VO_2_max 55.5 mL·kg^−1^·min^−1^: ♂ 58 20 ± 3.6 mL·kg^−1^·min^−1^; and ♀ 53 ± 4.5 mL·kg^−1^·min^−1^) completed the study experiment and were included in the analyses. Repeated measures ANOVA with optional post hoc Bonferroni correction was used to assess the association magnitude of changes in variables across the recovery methods. **Results:** All the investigated protocols were associated with significant changes in multiple recovery indices observed within all the investigated protocols (*p* ≤ 0.05). However, for this sample, they resulted in analogous effects on subjective variables, hormonal response, creatine kinase, CMJ parameters, and on-ice performance (between-protocol effect: *p* ≥ 0.002). Changes in creatine kinase were generally higher in males than females (*p* = 0.05), which might suggest that optimal recovery protocols in short-track are gender-dependent. **Conclusions:** Since compression and active cycling remain gold standard recovery protocols, a similar response from isocapnic breathing suggests it may be a modality particularly useful in real-world settings.

## 1. Introduction

Short-track speedskating is a dynamic Winter Olympic sport that involves multiple-day and multi-round competitions. The demanding nature of these events necessitates effective recovery between races. However, short intervals between efforts (approx. 20–30 min) may limit the potential of a skater’s regenerative abilities [1]. On the other hand, the specific position during speedskating and short-track mechanics, with its prolonged gliding phase and high intramuscular forces, may lead to muscle fatigue [2]. Fatigue in short-track speed skating could be linked to physiological changes, including increased deoxygenation and decreased blood flow to the muscles [3].

Optimizing recovery is crucial to prevent muscle damage, delayed-onset muscle soreness, and a decrease in subsequent performance that often occurs after high-intensity training and competition [4], particularly those involving anaerobic performance in speedskaters. Short-track lacks established recovery protocols, leading to various methods used in high-performance environments [2]. Low-intensity cycling-based active recovery (LIC) is a commonly applied recovery technique that may enhance the improvement of consecutive performance during intermittent exercise due to better lactate control and oxidative energy supply in subsequent efforts [5,6,7]. Another type of implemented recovery strategy is the application of compression garments, i.e., pneumatic compression boots (PCBs), that were proven effective in enhancing recovery from muscle damage [8]. However, reporting on the effectiveness of pneumatic methods between high-intensity efforts remains mixed [9,10,11]. Both methods are traditionally used by elite short-track athletes [2,12]. Given the high technical demands of short-track speedskating, alterations in optimal muscle tension and neuromuscular coordination may result in performance decline [13]. Noteworthy, both LIC and PCB techniques influence lower limbs and may affect muscle properties [14]; therefore, breathing-based recovery techniques might be a useful alternative.

The breathing-based recovery technique might utilize voluntary isocapnic hyperpnoea (VIH). The VIH technique is a common respiratory muscle-training method [15]. Notably, novel VIH applications for warm-up or recovery protocols were presented in the literature and demonstrated reduced blood lactate concentration [16,17]. This reduction in lactate is associated with improved repeated performance, suggesting that VIH can be a valuable tool for athletes. While the exact mechanisms underlying the VIH-induced decrease in blood lactate concentration remain unclear, it is evident that respiratory muscles play a crucial role in lactate metabolism [17,18,19]. Respiratory muscles, such as the diaphragm, can absorb lactate from the bloodstream during exercise, indicating their role as lactate consumers [20]. The diaphragm’s reliance on lactate for energy and its ability to mitigate adverse physiological responses suggest that VIH may improve performance by reducing fatigue and enhancing recovery [21].

The analysis of recent studies has shown a lack of standardized recovery protocols, limited research on various recovery techniques, and insufficient research on breathing-based recovery for short-track speedskating, highlighting the need for further investigation to optimize athlete performance and recovery. A better understanding of recovery processes might be beneficial for repeated sprint performance. It could help mitigate increased fatigue due to delayed-onset muscle soreness and acute muscle damage in short-track speedskaters. Therefore, we aimed to investigate the effectiveness of different recovery techniques in reducing fatigue in short-track speedskaters. The results of these investigations should help to optimize recovery strategies in short-track training and racing and improve performance during multi-round competitions. From our knowledge, this is also the first study that analyzed a group of elite speedskaters. Finally, we hypothesized that the breathing-based recovery technique may be particularly useful in short-track speedskating, as it is easy to apply and does not engage lower limbs.

## 2. Materials and Methods

This study was reviewed and approved by the Institute of Sport—National Research Institute Ethics Committee (approval no KEBN-23-90-KR). All procedures adhered to the Declaration of Helsinki. Informed written consent was obtained from all study participants. This study was designed as a crossover trial comparing the effects of three various recovery techniques (LIC, PCB, and VIH) in short-track speedskaters. All participants performed three testing sessions across two weeks. During each session, the participants performed two simulation efforts with the same recovery procedure after every effort. The sequence of the sessions was held in a random order. Before each session, the group training was standardized for 48 h, and the athletes were instructed not to perform any additional activities. Multiple fatigue-related indices were measured, including creatine kinase (CK) activity, cortisol (C) level, blood lactate (bLa) concentration, perceived pain and recovery, and countermovement jump (CMJ) height.

### 2.1. Participants

Fifteen elite junior short-track speedskaters (8 males and 7 females; age 18.3 ± 1.0 years, height 175.6 ± 7.5 cm, weight 73.7 ± 7.7 kg, 23.8 kg/m^2^, VO_2_max 55.5 mL·kg^−1^·min^−1^: ♂ 58 ± 3.6 mL·kg^−1^·min^−1^; and ♀ 53 ± 4.5 mL·kg^−1^·min^−1^) were included in this study. Participants were categorized into Tier 4 on the Participant Classification Framework [22], denoting highly trained athletes. The inclusion criteria were a valid medical certificate for speedskating competition, no prior experience with VIH, highly trained or elite performance status according to McKay et al. [22], and a minimum of 4 years of competitive athletic training. The exclusion criteria were any chronic or acute medical conditions within the preceding three months and ongoing medication use. The participants were recruited among development national team members from three different countries using convenience sampling [23]. We recruited 19 athletes; however, only 15 completed all the necessary measurements.

The G*Power software (version 3.1.9.2; Kiel University, Kiel, Germany) was used to estimate the required sample size based on a repeated measures analysis of variance (ANOVA) for between–within factors, an α of 0.05, a minimum expected effect size (Cohen’s f) of 0.5, and β of 0.95. A minimum sample size of 14 participants was required.

For group characteristics, the body mass was measured with the bioelectrical impedance analysis system Tanita BC-420MA (Tanita Corporation, Tokyo, Japan) in the morning before breakfast, body height was measured with the free-standing digital stadiometer seca 274 (seca GmbH & Co. KG, Hamburg, Germany), and maximum oxygen uptake was measured during a cycling ramp test to exhaustion using the Cortex Metamax B3 (Cortex Biophysik GmbH, Leipzig, Germany) and a Cyclus II Ergometer (RBM, Leipzig, Germany).

### 2.2. Experimental Procedures

#### 2.2.1. Measurements

**Figure 1:**
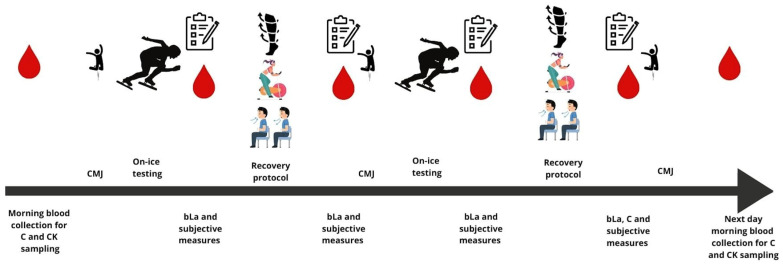
Design of one testing session, including blood sample testing (C—cortisol, CK—creatine kinase, bLa—blood lactate concentration), CMJ—countermovement jump, on-ice testing, and questionnaires assessing perceived pain and recovery.

The design of one testing session is presented in Figure 1 and thoroughly explained after.

Blood samples were collected multiple times: between 7:00 and 7:30 a.m., before breakfast on a testing day, on the next day, and during the testing sessions. Both CK and C were measured, as they are widely used to provide precise information about athletes’ muscle damage and stress and reflect the magnitude of the training stimulus [4]. CK was measured in the morning, both on the testing day and on the next day. C level was measured in the morning, both on the testing day and on the next day, and after the testing session. bLa was collected three and fifteen minutes after each on-ice effort with recovery protocol in between, as changes in bLa reflect the capillary supply and the muscle recovery [24]. All the blood parameters were measured in capillary blood samples (220 µL for creatine kinase activity and cortisol level analysis and 20 µL for blood lactate concentration analysis) taken from the fingertip. The samples for creatine kinase activity and cortisol level measurements underwent centrifugation at 5000 rpm for 10 min at a temperature of 4 °C. The CK and bLa analyses were performed immediately after the sampling. For C, the serum was aliquoted and stored at a temperature of −20 °C until the assays were performed during the next 24 h. All the sampling and analyses were performed by skilled laboratory technicians according to the manufacturer’s instructions. All the technicians were blinded regarding group allocation. Blood samples were measured by the following devices, i.e., CK activity via photometer lP 400 (Dr. Lange, Berlin, Germany), C level via Hoffmann-La Roche Ltd., Basel, Switzerland, with electrochemiluminescence (ECL) technology for assay analysis, and bLa concentration via Super GL2 analyzer (Dr. Muller Geratebau GmbH, Freital, Germany).

CMJ was reported to be the objective marker of fatigue and supercompensation and is widely used to monitor neuromuscular status in trained individuals [25]. In our study, CMJ was performed after the individual warm-up before the training session on ice and then in the 4th and 16th minutes after each testing effort, with the recovery protocol in between. Each time, all the participants performed CMJ barefoot 3 times. The jumping height and force were recorded. The highest value was included in further analysis. Chronojump-Boscosystem (Asociación Chronojump para la Investigación y Difusión de la Tecnología Aplicada a la Actividad Física y el Deporte, Valencia, Spain) dynamometric platforms with accessory software (version 2.3.0-63) were used. The measurements were performed by an experienced strength and conditioning coach. All the participants were familiarized with CMJ and the testing protocol, as they performed the measurements as a part of their daily routine for many months.

To assess the influence of investigated protocols, the athletes described their perceived recovery and pain 3 and 15 min after cessation of exercise on 1–10 scales. Subjective Perceived Recovery Status Scale (PRSS) and Numeric Rating Pain Scale (NRPS) were applied, as both were already validated and applied in the athletic settings [26,27].

#### 2.2.2. Exercise Procedure

On-ice testing was performed on an oval ice track with a length of 111.111 m, located at sea level (142 m asl). All the athletes performed a standardized, split 1000 m race simulation, and the average lap time (in seconds) was used for further analyses. ProChip Timing System and accessory software (version 5.1; MYLAPS Sports Technology, Haarlem, The Netherlands) were applied for time measurements. The track was cleaned before each testing effort.

#### 2.2.3. Recovery Procedures

The LIC recovery protocol consisted of a 10 min ride guided by a subjective rate-of-perceived (RPE) effort. The participants were instructed to maintain RPE 2–3 on the CR10 Borg Scale [28] and cadence in the 75–95 revolutions per minute range. All the athletes were familiar with their aerobic threshold heart rate. To ensure the low intensity of effort, the participants were advised not to exceed this value even if the RPE suggested riding with higher intensity. Wattbike Air (Wattbike Ltd., Nottingham, UK) indoor cycling trainers were used.

The PCB protocol consisted of 10′ of lying down while using the Aerify Charge 2.0 Recovery Boots system (Aerify Recovery Technology, Riga, Latvia). The device was set in “Active Recovery” mode and to “Intensity Level 10”. All the participants used the devices with the appropriate size.

The VIH recovery protocol consisted of two 4 min intervals of purposeful and energetic breathing with a 20 breaths·min^−1^ frequency with a 2 min break. The participants were instructed to use diaphragmatic breathing patterns and minimize upper shoulder and chest movements. The Isocapnic BreathWayBetter devices (Isocapnic Technologies Inc., Kelowna, BC, Canada) with 6 L bags were applied. The manufacturer’s software was used to provide visual guidance to the participants. The protocol was performed in a seating position, under the supervision of a qualified physiotherapist.

### 2.3. Statistical Analysis

Data analysis was performed using SPSS statistical software (version 25.0, SPSS Inc., Chicago, IL, USA). Mean values ± standard deviation (SD) were calculated for relevant parameters. To assess normality, Shapiro–Wilk tests were employed. Levene’s test was used to verify the homogeneity of variance. All analyzed parameters exhibited normal distribution and equal variance. Repeated-measures ANOVA (RM-ANOVA) was conducted to examine the effects of time, sex, and recovery protocol on biochemical markers, pain and recovery scales, and CMJ height and power. In cases of significant interaction effects, Bonferroni post hoc tests were employed with a significance level of *p* = 0.006. Partial eta-squared (η^2^) was calculated to estimate the effect size, categorized as small (0.02 < η^2^ < 0.049), moderate (0.05 < η^2^ < 0.79), or large (η^2^ ≥ 0.8) [29]. For all statistical tests except post hoc comparisons, a significance level of *p* ≤ 0.05 was adopted.

## 3. Results

**Figure 2:**
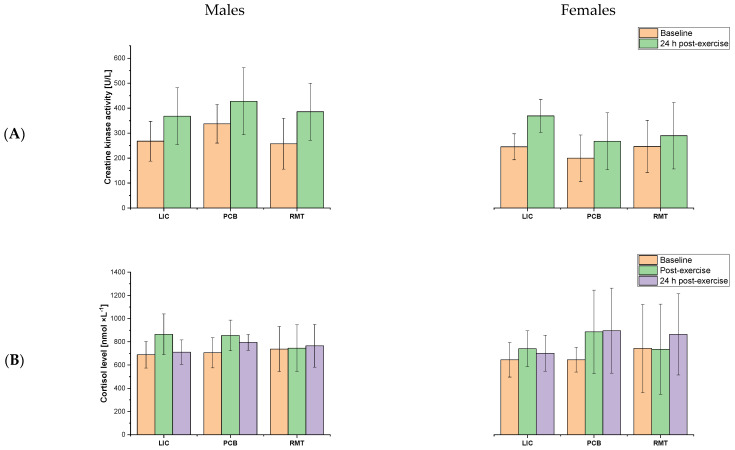

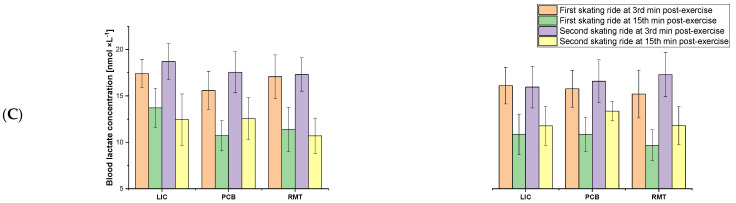
Mean ± SD of biochemical markers, i.e., (**A**) creatine kinase activity [U/L], (**B**) cortisol level [nmol × L^−1^], and (**C**) blood lactate concentration [nmol × L^−1^] at different timelines after speedskating. Abbreviations: LIC—low-intensity cycling, PCB—pneumatic compression boots, VIH—voluntary isocapnic hyperpnoea.

Figure 2 shows the mean ± SD of the biochemical markers. The RM-ANOVA revealed a significant moderate effect of time (*F*_1,38_  =  107.4, *p*  ≤  0.001, η^2^  =  0.74) and sex (*F*_1,38_  =  8.8, *p*  = 0.005, η^2^  =  0.19) for creatine kinase activity, a significant moderate effect of *time* (*F*_2,78_  =  9.7, *p*  ≤  0.001, η^2^  =  0.20) for cortisol level, and a significant large effect of *time* (*F*_3,75_  =  122.5, *p*  ≤  0.001, η^2^  =  0.85) for blood lactate concentration. Moreover, an interaction effect of time  ×  sex (*F*_1,38_  =  4.0, *p*  =  0.05, η^2^  =  0.09) for CK activity was found. The creatine kinase activity increased post-exercise despite the recovery procedures in both males and females, compared to baseline (*p* ≤ 0.001). Finally, the increase in creatine kinase activity was greater in males than in females (*p* = 0.005). The cortisol level increased immediately post-exercise and on the next day was significantly higher compared to the morning on a testing day for all the recovery procedures (*p* = 0.003 and *p* = 0.005, respectively). The blood lactate concentration increased after the second skating ride at the 3rd minute post-exercise (*p* ≤ 0.001), followed by a decrease at the 15th minute post-exercise (*p* ≤ 0.001).

**Figure 3:**
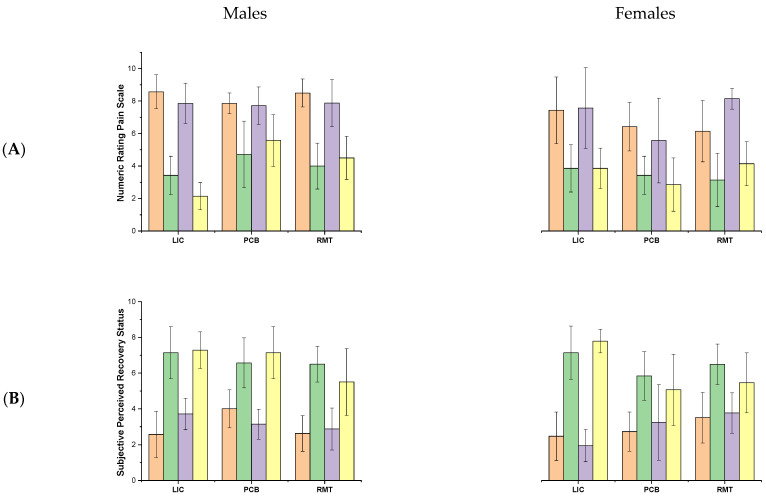
Mean ± SD of questionnaire assessment, i.e., (**A**) Numeric Rating Pain Scale and (**B**) Subjective Perceived Recovery Status Scale at the 3rd and 15th minutes post-exercise and after recovery procedures. Abbreviations: LIC—low-intensity cycling, PCB—pneumatic compression boots, VIH—voluntary isocapnic hyperpnoea. The orange indicates the first skating ride at 3rd min post-exercise, the green represents the first skating ride at 15th min post-exercise, the purple represents second skating ride at 3rd min post-exercise and the yellow represents second skating ride at 15th min post-exercise.

The Numeric Rating Pain Scale (NRPS) and Subjective Perceived Recovery Status Scale (PRSS) were collected at the 3rd and 15th minutes post-exercise, and the recovery procedures were applied between the measurements. The RM-ANOVA revealed a large significant effect of *time* (*F*_1,75_  =  175.5, *p*  ≤  0.001, η^2^  =  0.83) and a moderate significant effect of *sex* (*F*_1,75_  =  9.0, *p*  = 0.008, η^2^  =  0.18) for PRSS, as well as a large significant effect for *time* (*F*_1,75_  =  185.8, *p*  ≤  0.001, η^2^  =  0.83) for NRPS. However, no interaction effect was calculated for this part of the analysis. The post hoc analysis showed a decrease in NPRS after all the recovery protocols after both testing efforts (*p* ≤ 0.001) and a greater decrease in NRPS in males than females after the first testing effort (*p* ≤ 0.001). On the other hand, the PRSS increased after all the recovery protocols after both testing efforts (*p* ≤ 0.001) (Figure 3).

**Figure 4:**
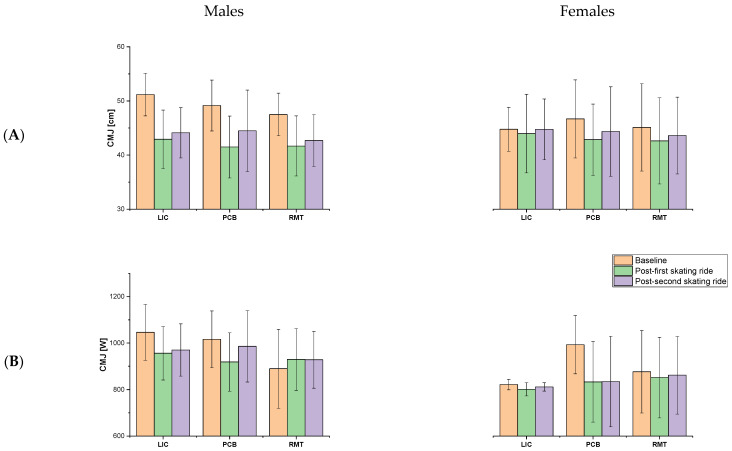
Mean ± SD of countermovement jump height in centimeters (**A**) and power in watts (**B**) at baseline and twice after the testing efforts. Abbreviations: LIC—low-intensity cycling, PCB—pneumatic compression boots, VIH—voluntary isocapnic hyperpnoea.

The CMJ height and power were collected at baseline and twice after recovery protocols. The RM-ANOVA revealed a significant moderate main effect of *time* (*F*_2,75_  =  62.3, *p*  ≤  0.001, η^2^  =  0.66) for height, as well as of *time* (*F*_2,75_  =  27.3, *p*  ≤  0.001, η^2^  =  0.45) and *sex* (*F*_1,35_  =  7.3, *p*  =  0.011, η^2^  =  0.18) for power. Moreover, a significant moderate interaction effect of *time* × *sex* (*F*_2,75_  =  4.7, *p*  =  0.013, η^2^  =  0.12) for power was found. The post hoc analysis showed a decrease in CMJ height after all the recovery protocols after both testing efforts (*p* ≤ 0.001). Moreover, males reached greater CMJ power than females both at baseline and after the second skating ride (*p* = 0.001 and *p* = 0.006, respectively) (Figure 4).

## 4. Discussion

We aimed to investigate the effect of several recovery protocols among elite speedskaters. This study showed: (a) the influence of different recovery procedures on psychophysiological outcomes in elite speedskating, (b) evaluated post-exercise alterations in physiological outcomes, and (c) assessed the effect of various recovery procedures on developing fatigue, expressed by biochemical markers (CK, C, bLa), subjective pain sensitivity, recovery assessment, performance outcomes, and neuromuscular status.

In summary, we observed significant increases in muscle damage and stress markers after race-specific exercise, expressed by changes in CK, C, and bLa. There were also variations in perceived pain and recovery across different strategies and reductions in performance metrics following race-specific on-ice efforts. Moreover, in this study we found several significant differences between male and female athletes among their recovery markers. The main point was related to significant sex differences in CK activity, with males exhibiting greater increases after exercises compared to females. This may suggest that muscle damage contributes to overall fatigue more in males compared to females. Our findings are in line with general knowledge of higher muscle fatigue resistance in females [30]. However, the observed differences may originate from higher absolute mechanical work performed by males, as they achieve higher speeds, contraction forces, and tension [31]. No significant differences between the investigated protocols were found. Therefore, it suggests that a difference between the effects of recovery protocols we investigated is negligible from the practical point of view.

The presented results highlighted the importance of optimizing recovery strategies to mitigate fatigue and enhance performance in elite speed skating. This complex sport based on repeated racing efforts creates specific challenges in terms of recovery and poses an interesting challenge to sports scientists. Intermittent efforts or repeated racing are common in multiple team (i.e., football, basketball, or hockey) and individual sports (i.e., fencing or swimming) [32,33,34]. Despite this, it should be noted that the available literature is limited in presenting evidence concerning recovery procedures and strategies in short-track speedskaters [18,35]. These studies investigated breathing-based recovery techniques, including VIH and hot water therapy. Previous research has shown that VIH may be an effective recovery protocol for elite athletes following anaerobic exercise [18]. Additionally, hot-water therapy could induce long-term benefits on maximal isometric strength without compromising aerobic or anaerobic adaptations or field performance [35]. However, the feasibility of this protocol might be limited in real-world settings, i.e., between races during the event. Therefore, whereas cycling-based active recovery or compression devices remain popular choices in short-track, VIH might constitute an interesting addition or alternative, as it leads to analogous effects in the study participants.

Noteworthily, Chiappa et al. observed improved post-exercise bLa clearance with inspiratory loading, attributing it to increased lactate uptake by the respiratory muscles and heart [36,37]. The more pronounced difference in bLa between inspiratory loading and passive recovery during the initial 5 min of recovery suggests a faster bLa decline rate compared to traditional recovery methods relying on skeletal muscle engagement [36]. This implies that VIH recovery protocols might be particularly beneficial in scenarios with limited time between consecutive efforts. Brown et al. also reported that loading trained inspiratory muscles accelerates lactate recovery kinetics [17]. In our study, there were 12 min between blood sampling for bLa, which might have evened possible differences in bLa kinetics between the investigated protocols. The issue requires further investigation, and its practical usefulness might depend on the competition schedule and recovery interval between the subsequent races.

Breathwork recovery protocols represent a relatively unexplored domain [18,38]. Our study not only shed light on this phenomenon but also underscored the necessity for additional research. Scarce findings suggest that breathing protocols may enhance autonomic changes, i.e., increase heart rate variability and respiratory sinus arrhythmia, and modify the activity of the nervous and endocrine systems [38,39]. Possible psychological outputs might include increased comfort, relaxation, vigor, and alertness, and reduced stress, anxiety, or anger [21,38]. Future inquiries could encompass breathwork protocols varying in coordination, intensity, and duration while also expanding the monitoring of stress- and fatigue-related indicators. Alternatively, investigations could delve deeper into explaining the mechanisms underlying the impact of breathwork and respiratory muscle training on psychophysiological recovery. Finally, investigating the studied recovery protocols in real-life competition settings, with different populations or sport-specific contexts, may guide best practices in applied sports science.

### Strengths and Limitations

The distinctive cohort of top-tier athletes and the applied relevance of the findings may be seen as strengths of this study. Additionally, we monitored a wide array of parameters, offering a comprehensive insight into athletes’ recovery from fatigue. However, the presented research is not free from limitations. The small sample size is one of them. This study exclusively focused on speedskaters, which facilitated control over confounding variables but also hindered the generalizability of the findings. Moreover, there was no control group that was restricted from performing any recovery protocols. Finally, the participants were not blinded and had many opportunities to communicate with each other across the testing period, which might have introduced bias into their perception of subjective pain and recovery measures.

## 5. Conclusions

This study examined the impact of LIC, PCB, and VIH on elite speedskaters. In the investigated sample, there were no statistically significant differences between the investigated protocols. Sex differences were observed in changes in muscle damage markers. and performance. This might suggest that optimal recovery protocols in short-track are gender-dependent, as muscle damage is higher in males compared to females. Since compression and active cycling remain gold standard recovery protocols, a similar response from isocapnic breathing suggests it may be a modality particularly useful in real-world settings. Further research is needed to fully understand the benefits and limitations of each recovery protocol, especially VIH.

## Data Availability

Data will be made available upon reasonable request to the corresponding authors.

## References

[1] Bullock N., Martin T.D., Zhang A. (2008). Performance analysis of world class short track speed skating: What does it take to win?. Int. J. Perform. Anal. Sport.

[2] Hettinga F.J., Konings M.J., Cooper C.E. (2016). Differences in Muscle Oxygenation, Perceived Fatigue and Recovery between Long-Track and Short-Track Speed Skating. Front. Physiol..

[3] Rundell K.W. (1996). Compromised oxygen uptake in speed skaters during treadmill in-line skating. Med. Sci. Sports Exerc..

[4] Lee E.C., Fragala M.S., Kavouras S.A., Queen R.M., Pryor J.L., Casa D.J. (2017). Biomarkers in Sports and Exercise: Tracking Health, Performance, and Recovery in Athletes. J. Strength Cond. Res..

[5] Mika A., Mika P., Fernhall B., Unnithan V.B. (2007). Comparison of recovery strategies on muscle performance after fatiguing exercise. Am. J. Phys. Med. Rehabil..

[6] Fujita Y., Koizumi K., Sukeno S., Manabe M., Nomura J. (2009). Active recovery effects by previously inactive muscles on 40-s exhaustive cycling. J. Sports Sci..

[7] Koizumi K., Fujita Y., Muramatsu S., Manabe M., Ito M., Nomura J. (2011). Active recovery effects on local oxygenation level during intensive cycling bouts. J. Sports Sci..

[8] Hill J., Howatson G., van Someren K., Leeder J., Pedlar C. (2014). Compression garments and recovery from exercise-induced muscle damage: A meta-analysis. Br. J. Sports Med..

[9] O’Donnell S., Driller M.W. (2015). The effect of intermittent sequential pneumatic compression on recovery between exercise bouts in well-trained triathletes. J. Sci. Cycl..

[10] Overmayer R.G., Driller M.W. (2018). Pneumatic Compression Fails to Improve Performance Recovery in Trained Cyclists. Int. J. Sports Physiol. Perform..

[11] Wisniowski P., Cieslinski M., Jarocka M., Kasiak P.S., Makaruk B., Pawliczek W., Wiecha S. (2022). The Effect of Pressotherapy on Performance and Recovery in the Management of Delayed Onset Muscle Soreness: A Systematic Review and Meta-Analysis. J. Clin. Med..

[12] Yang C., Xu Y., Yang Y., Xiao S., Fu W. (2020). Effectiveness of Using Compression Garments in Winter Racing Sports: A Narrative Review. Front. Physiol..

[13] Konieczny M., Matuska J., Pakosz P., Domaszewski P., Skulska M., Herrero P., Skorupska E. (2024). Resting muscle tension and trigger points in elite junior short-track athletes and healthy non-athletes: A cross-sectional examination. Front. Sports Act. Living.

[14] Chleboun G.S., Howell J.N., Baker H.L., Ballard T.N., Graham J.L., Hallman H.L., Perkins L.E., Schauss J.H., Conatser R.R. (1995). Intermittent pneumatic compression effect on eccentric exercise-induced swelling, stiffness, and strength loss. Arch. Phys. Med. Rehabil..

[15] Kowalski T., Granda D., Klusiewicz A. (2024). Practical Application of Respiratory Muscle Training in Endurance Sports. Strength Cond. J..

[16] Romer L.M., McConnell A.K., Jones D.A. (2002). Effects of inspiratory muscle training upon recovery time during high intensity, repetitive sprint activity. Int. J. Sports Med..

[17] Brown P.I., Sharpe G.R., Johnson M.A. (2008). Inspiratory muscle training reduces blood lactate concentration during volitional hyperpnoea. Eur. J. Appl. Physiol..

[18] Kowalski T., Wilk A., Rebis K., Lohse K.M., Sadowska D., Klusiewicz A. (2024). Influence of voluntary isocapnic hyperpnoea on recovery after high-intensity exercise in elite short-track speedskaters—Randomized controlled trial. BMC Sports Sci. Med. Rehabil..

[19] Spengler C.M., Roos M., Laube S.M., Boutellier U. (1999). Decreased exercise blood lactate concentrations after respiratory endurance training in humans. Eur. J. Appl. Physiol. Occup. Physiol..

[20] Fregosi R.F., Dempsey J.A. (1986). Effects of exercise in normoxia and acute hypoxia on respiratory muscle metabolites. J. Appl. Physiol..

[21] Hopper S.I., Murray S.L., Ferrara L.R., Singleton J.K. (2019). Effectiveness of diaphragmatic breathing for reducing physiological and psychological stress in adults: A quantitative systematic review. JBI Database Syst. Rev. Implement. Rep..

[22] McKay A.K.A., Stellingwerff T., Smith E.S., Martin D.T., Mujika I., Goosey-Tolfrey V.L., Sheppard J., Burke L.M. (2022). Defining Training and Performance Caliber: A Participant Classification Framework. Int. J. Sports Physiol. Perform..

[23] Sedgwick P. (2013). Convenience sampling. BMJ.

[24] Tesch P.A., Wright J.E. (1983). Recovery from short term intense exercise: Its relation to capillary supply and blood lactate concentration. Eur. J. Appl. Physiol. Occup. Physiol..

[25] Claudino J.G., Cronin J., Mezencio B., McMaster D.T., McGuigan M., Tricoli V., Amadio A.C., Serrao J.C. (2017). The countermovement jump to monitor neuromuscular status: A meta-analysis. J. Sci. Med. Sport.

[26] Laurent C.M., Green J.M., Bishop P.A., Sjokvist J., Schumacker R.E., Richardson M.T., Curtner-Smith M. (2011). A practical approach to monitoring recovery: Development of a perceived recovery status scale. J. Strength Cond. Res..

[27] Williamson A., Hoggart B. (2005). Pain: A review of three commonly used pain rating scales. J. Clin. Nurs..

[28] Williams N. (2017). The Borg Rating of Perceived Exertion (RPE) scale. Occup. Med..

[29] Richardson J.T.E. (2011). Eta squared and partial eta squared as measures of effect size in educational research. Educ. Res. Rev..

[30] Hicks A.L., Kent-Braun J., Ditor D.S. (2001). Sex Differences in Human Skeletal Muscle Fatigue. Exerc. Sport Sci. Rev..

[31] Billaut F., Bishop D. (2009). Muscle fatigue in males and females during multiple-sprint exercise. Sports Med..

[32] Spencer M., Bishop D., Dawson B., Goodman C. (2005). Physiological and metabolic responses of repeated-sprint activities:specific to field-based team sports. Sports Med..

[33] Hinzpeter J., Zamorano A., Cuzmar D., Lopez M., Burboa J. (2014). Effect of active versus passive recovery on performance during intrameet swimming competition. Sports Health.

[34] Milia R., Roberto S., Pinna M., Palazzolo G., Sanna I., Omeri M., Piredda S., Migliaccio G., Concu A., Crisafulli A. (2014). Physiological responses and energy expenditure during competitive fencing. Appl. Physiol. Nutr. Metab..

[35] Meline T., Solsona R., Antonietti J.P., Borrani F., Candau R., Sanchez A.M. (2021). Influence of post-exercise hot-water therapy on adaptations to training over 4 weeks in elite short-track speed skaters. J. Exerc. Sci. Fit..

[36] Chiappa G.R., Roseguini B.T., Alves C.N., Ferlin E.L., Neder J.A., Ribeiro J.P. (2008). Blood lactate during recovery from intense exercise: Impact of inspiratory loading. Med. Sci. Sports Exerc..

[37] Stanley W.C. (1991). Myocardial lactate metabolism during exercise. Med. Sci. Sports Exerc..

[38] Zaccaro A., Piarulli A., Laurino M., Garbella E., Menicucci D., Neri B., Gemignani A. (2018). How Breath-Control Can Change Your Life: A Systematic Review on Psycho-Physiological Correlates of Slow Breathing. Front. Hum. Neurosci..

[39] Kowalski T., Obminski Z., Walerianczyk W., Klusiewicz A. (2025). The acute effect of respiratory muscle training on cortisol, testosterone, and testosterone-to-cortisol ratio in well-trained triathletes—Exploratory study. Respir. Physiol. Neurobiol..

